# *In situ* repair or reconstruction of the abdominal aorta-iliac artery by autologous fascia-peritoneum with posterior rectus sheath for the treatment of the infected abdominal aortic and iliac artery aneurysms: A case series and literature review

**DOI:** 10.3389/fcvm.2022.976616

**Published:** 2022-11-08

**Authors:** Lubin Li, Guolong Liu, Benxiang Yu, Wenqiang Niu, Zhigang Pei, Juwen Zhang, Haijie Che, Fubo Song, Mu Yang

**Affiliations:** Department of Vascular Surgery, Yantai Yuhuangding Hospital, Yantai, China

**Keywords:** infected aneurysm, autologous fascia-peritoneum with posterior rectus sheath, *in situ*, repair, reconstruction

## Abstract

**Background:**

Infected abdominal aortic and iliac artery aneurysms are considered acute and severe diseases with insidious onset, rapid development, and high mortality in vascular surgery. Currently, there is no better treatment, either anatomic or extra-anatomical repair.

**Case presentation:**

From February 2018 to April 2022, 7 patients with infected abdominal aortic and iliac artery aneurysms did not have sufficient autologous venous material for repair. With the consent of the Ethics Committee of the hospital, it uses the autologous peritoneal fascial tissue with rectus sheath to repair or reconstruct the infected vessels *in situ*. There were 5 cases of infected abdominal aortic aneurysm, 1 case of an infected common iliac aneurysm, and 1 case of the infected internal iliac aneurysm. Aortoduodenal fistula was found in 3 cases, all of them were given duodenal fistula repair and gastrojejunostomy and cholecystostomy. Three cases of infected abdominal aortic aneurysms were repaired with the autologous peritoneal fascial tissue patch, and 2 cases of infected abdominal aortic aneurysms were reconstructed by the autologous peritoneal fascial tissue suture to bifurcate graft *in situ*, the autologous peritoneal fascial tissue suture reconstructed the rest 2 cases of infected iliac aneurysm to tubular graft *in situ*. It was essential that Careful debridement of all infected tissue and adequate postoperative irrigation and drainage. Antibiotics were administered perioperatively, and all patients were subsequently treated with long-term antibiotics based on bacterial culture and susceptibility results of infected tissues and blood. All 7 patients had underwent surgery successfully. But there were 2 cases died of anastomotic infection or massive hemorrhage after the operation, the other 5 cases survived. The follow-up time was 2–19 months. The enhanced CT of postoperation showed that the reconstructed arteries were smooth without obvious stenosis or expansion, and no abdominal wall hernia occurred.

**Conclusion:**

*In situ* repair or reconstruction with autologous peritoneal fascial tissue with rectus sheath is a feasible treatment for the infected aneurysm patients without adequate autologous venous substitute, but it still needs long-term follow-up and a large sample to be further confirmed.

## Introduction

Infected aneurysm is an uncommon but fatal disease in the general population, with an incidence of 0.8–2% reported in the literature (1). Its most common site is the abdominal aorta, especially in the lower renal segment, followed by the thoracic aorta and iliac arteries. Some literatures have also reported infectious aneurysms of the coronary arteries, carotid arteries, and popliteal arteries (2–4). The classic triad of symptoms of infectious aneurysms is fever, local pain, and pulsatile mass. However, most patients often present with non-specific symptoms. Some patients with infectious aneurysms of the abdominal aorta and iliac arteries may present with abdominal pain, back pain, and infection, while others are not diagnosed until severe sepsis or aneurysm rupture occurs. Infected aneurysms, regardless of the location, are usually irreversible and have a high chance of rupture and mortality. Complete removal of the infected tissue along with *in situ* or extra-anatomic revascularization is recommended by current guidelines. Some papers have also reported effective cases using *in situ* reconstruction or endovascular aneurysm exclusion. Autologous veins, such as great saphenous vein and superficial femoral vein, are preferred as revascularization materials. However, some patients have insufficient autologous veins, such as those with varicose veins and venous insufficiency of the lower extremities.

We herein report 7 patients with infective abdominal aortic and iliac aneurysms who were treated in the past 4 years. All 7 patients were diagnosed with infected aneurysms based on blood culture and intraoperative infected tissue culture. After consulting the relevant literature, we employed autologous fascia-peritoneum with a posterior rectus sheath to repair or reconstruct blood supply *in situ* to treat infectious aneurysms.

## Case presentation

From February 2018 to April 2022, 7 patients (5 men and 2 women, age: 60–74 years, mean age: 67.3 years) with infectious abdominal aortic and iliac aneurysms were treated with autologous fascia-peritoneum with a posterior rectus sheath. The chief complaint was fever with lower back and abdominal pain. The onset time varied from 1 day to 1 year (mean: 62.0 days). The sites of infection were abdominal aorta in 5 cases and iliac artery in the other 2 cases. In addition, diabetes mellitus was present in 4 cases, while hepatobiliary infectious disease was present in 3 cases (Table 1). The operation and prognosis are listed in Table 2.

**Table 1:** General information of patients.

**Case#**	**Age**	**Gender**	**Clinical manifestations**	**Onset time**	**Concomitant disease**	**Past medical/surgical history**	**Diagnosis**	**Infected site and aneurysm size**
#1	60	M	Low back and abdominal pain	1 d	HP	Livestock history (sheep)	Infected abdominal aortic pseudoaneurysm	The maximum section of the lower abdominal aorta was about 3.4*2.6 cm
#2	69	M	Low back pain	1 m	HP	N	Infected abdominal aortic pseudoaneurysm	The maximum section of the lower abdominal aorta was about 4.3*4.7 cm
#3	67	F	Abdominal pain with fever	1 y	HP, DM, CAD, SS	History of EVAR (2 years ago), pneumonia (*Klebsiella pneumoniae*)	AAA stent infection	The maximum section of the lower abdominal aorta was about 4.6*5.3 cm
#4	62	M	Low back pain	22 d	HP, DM	History of liver abscess (prior to 1 month)	Infected abdominal aortic pseudoaneurysm	The maximum section of the lower abdominal aorta was about 7.0 * 6.5 cm
#5	74	F	Periumbilical pain with fever	7 d	HP, DM, CAD	N	Infected abdominal aortic pseudoaneurysm	The maximum section of the lower abdominal aorta was about 6.2*7.8 cm
#6	77	M	Fever with severe left flank pain	2 d	Cholelithiasis	Laparoscopic cholecystectomy, left hepatic lobectomy + choledochotomy + choledochojejunostomy	Infected left iliac aneurysm rupture	The maximum section of LCIA was about 5.8 * 5.4 cm
#7	62	M	Abdominal pain	7 d	DM	Gallstone	Infected left iliac pseudoaneurysm	The maximum section of LCIA was 5.6 * 6.0

M, Male; F, Female; d, day; m, month; y, year; HP, hypertension; DM, Diabetes mellitus; CAD, coronary atherosclerotic heart disease; SS, Sjogren Syndrome; AAA, abdominal aortic aneurysm; LCIA, Left common iliac arte.

**Table 2:** Perioperative and prognosis of patients.

**Case#**	**Preoperative culture results**	**Timing of surgery**	**Surgical method**	**Autograft shape**	Autograft size (length *width/diameter *length)	**Aorto-gastrointestinal fistula**	**Infected tissue culture**	**Postoperative complications**	**ICU time**	**Hospital stay**	**Follow-up time**	**Prognosis**	**Cause of death**
#1	*Brucella*	Limited procedure	Autologous fascia-peritoneum with posterior rectus sheath repair	Patch	1.0*3.0 cm	N	*Brucella*	No	1 d	39 d	29 m	Discharge	
#2	*Salmonella*	Limited procedure	Reconstruction of abdominal aorta and iliac artery with autologous fascia-peritoneum with posterior rectus sheath repair	Bifurcated tubular	1.5*12.0 cm, 1.0*5.0 cm	N	*Salmonella*	Renal dysfunction (recovered 6 days after CVVH)	10 d	40 d	23 m	Discharge	
#3	Multidrug-resistant *Escherichia coli*	Limited procedure	Stent graft removal, autologous fascia-peritoneum with posterior rectus sheath repair + duodenal fistula repair, gastrojejunostomy, cholecystostomy	Patch	2.0*2.0 cm	Y	Large intestine	No	2 d	58 d	15 m	Discharge	
#4	*Klebsiella pneumoniae*	Limited procedure	Autologous fascia-peritoneum with posterior rectus sheath repair + enterostomy	Patch	2.0*4.0 cm	Y	*Klebsiella pneumoniae*	Aortic repair site infection major bleeding	2 d	83 d	N	Death	Major bleeding at the aortic repair site 12 days after surgery
#5	*Salmonella*	Emergency surgery	Emergency EVAR for rupture; 12 days later, open stent removal, Reconstruction of abdominal aorto-iliac autologous fascia-peritoneum with posterior rectus sheath repair + duodenal fistula repair, gastrojejunostomy, and cholecystostomy	Bifurcated tubular	2.0*8.0 cm, 1.3*3.0 cm	Y	*Salmonella*, Large intestine	Gastrointestinal fistula, aortic anastomotic leakage	14 d	66 d	N	Death	Aortic anastomotic infection and massive hemorrhage 7 d after operation
#6	N	Emergency surgery	Reconstruction of left common iliac-external iliac artery autologous fascia-peritoneum with posterior rectus sheath repair	Tubular	1.0*5.0 cm	N	*Enterococcus faecium*	Gastroparesis, dysbacteriosis	3 d	24 d	19 m	Discharge	
#7	*Staphylococcus aureus*	Emergency surgery	Reconstruction of left common iliac-external iliac artery autologous fascia-peritoneum with posterior rectus sheath	Tubular	0.8*10.0 cm	N	*Staphylococcus aureus*	N	1 d	17 d	3 m	Discharge	

d, day; m, month; CVVH, continuous veno-venous hemofiltration.

**Figure 1:**
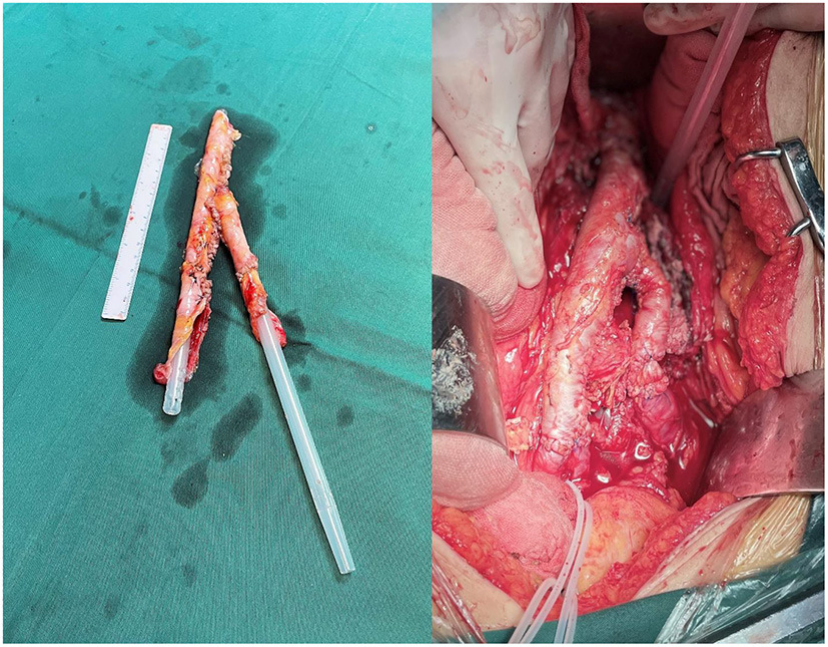
Autologous fascia-peritoneum with a posterior rectus sheath was sutured for bifurcated tubular.

Elective surgery was performed in 4 cases. In two cases (Cases 1 and 2), the site of the disease was the lower abdominal aorta. Among them, in case 1, a localized protrusion about 2 cm above the level of the rectus abdominis bifurcation was observed during surgery, severe adhesion between the duodenum and the inferior vena cava, necrotic infected tissue around the abdominal aorta was removed, a break was found after trimming the abdominal aortic wall, and the anterior wall of the abdominal aorta was repaired *in situ* using autologous peritoneal-fascial tissue with the posterior rectus sheath as a patch; in case 2, due to the wide range of abdominal aortic infection and severe adhesion of the surrounding tissue, infectious abdominal aortic aneurysm resection was decided during surgery. A bifurcated tubular *in situ* reconstruction of the abdominal aorta-bilateral iliac arteries were made using autologous peritoneal-fascial tissue suture with the posterior rectus sheath (Figure 1). The other 2 cases (Cases 3 and 4) were found to have abdominal aorta-duodenal fistulas, one of which (Case 3) had previously undergone EVAR at another hospital, during which significant stent exposure was observed after removing the necrotic tissue around the abdominal aorta. In contrast, a fistula was observed at the horizontal duodenum; after freeing the abdominal aortic fistula, the abdominal aortic stent graft was removed to remove the local arterial wall adhered to the abscess and edematous and to avoid stenosis and tear after the abdominal aortic suture, the infected abdominal aorta was reconstructed *in situ* using autologous peritoneal-fascial tissue with the posterior rectus sheath of the rectus abdominis muscle, duodenal fistula repair, gastrojejunostomy, and cholecystostomy. In the other case (Case 4), after local debridement of the abdominal aorta at the site of infection, the horizontal part of the duodenum was invaded and ulcerated by the aneurysm. The pseudoaneurysm tumor was filled with a large amount of thrombus, necrotic material, and pus, which was completely removed and sent for bacterial culture. Exploration showed large damage of about 2 × 4 cm in the left wall of the middle abdominal aorta, and an autologous peritoneal-fascial tissue patch with a posterior rectus sheath was used to repair the defective abdominal aorta, followed by duodenal fistula repair and enterostomy.

In one case (case 6), EVAR was performed in the emergency department for rupture during hospitalization for anti-infection (day 39 after hospitalization). In this case, postoperative infection control was difficult (persistent fever, abdominal pain). Laparotomy was performed 12 days after surgery; during the operation, it was found that the posterior wall of the horizontal duodenum ruptured and communicated with the abdominal aortic aneurysm. The aneurysm cavity was filled with pus and necrotic material, and covered stents were observed. Hence, stent graft removal, abdominal aorto-bilateral iliac artery resection, and *in situ* reconstruction of the abdominal aorto-bilateral iliac artery + duodenal fistula repair using autologous fascia-peritoneum with a posterior rectus sheath to suture into a bifurcated tube, gastrojejunostomy, and cholecystostomy were performed.

An ultrasound evaluation of the deep and superficial veins of the lower extremities was carried out for all the above 5 patients underwent during their hospitalization. Of these, 2 patients had varicose veins of the lower extremities and 1 had a deep venous valve insufficiency, considering that the patient had venous insufficiency of the lower extremities and did not have sufficient autologous veins to achieve vascular reconstruction, peritoneal-fascial tissue with the posterior rectus sheath was selected for vascular reconstruction. During our postoperative follow-up, it was found that the application of tissue to reconstruct the vascular morphology was good, so after communicating with the patient and his family in Case 3, we still selected the peritoneal-fascial tissue with the posterior rectus sheath for vascular reconstruction. In Case 2, due to the wide range of abdominal aortic infections more walls of the infected abdominal aorta were removed, the length of the vein required to reconstruct the arterial vessels was long, the damage to the lower limbs was large, and adequate caliber could not be ensured for vascular reconstruction. After communicating with the family, we selected the peritoneal-fascial tissue suture “Y” tubular structure with the posterior rectus sheath for vascular reconstruction.

There were 2 cases (cases 6 and 7) of emergency surgery for ruptured infectious aneurysms. Both patients had infected left iliac aneurysms and underwent aneurysm resection, removal of necrotic tissue, tubular reconstruction of the common iliac-external iliac artery *in situ* using autologous fascia-peritoneum with a posterior rectus sheath, suture of the distal internal iliac artery, and irrigation and drainage of the infected lesion. Because the procedure was urgent, the patient's leg veins could not be assessed to ensure smooth operation, and we did not use autologous vein vessels.

In all operations, the necrotic and infected tissue in the infected lesion was completely removed and 2–4 drainage tubes were placed to facilitate postoperative irrigation and drainage.

Five cases were found to be positive for bacterial culture. Therefore, sensitive antibiotics were selected for treatment based on the drug sensitivity test results. The infected tissue was used for bacterial culture, and sensitive antibiotics were used for treatment according to the culture susceptibility results. Bacterial culture results are detailed in Table 2.

The success rate of operation was 100%. The length of hospital stay was 17–83 days. The length of ICU stay was 1–14 days. One case showed postoperative renal insufficiency, which gradually improved after 6 days of paraclinoid hemofiltration. One case had dysbacteriosis, which was improved after adjusting antibiotics and probiotics. Two patients died during hospitalization owing to postoperative massive hemorrhage of the abdominal aortic repair site, which occurred on the 7 and 12th day after operation, respectively. The remaining 5 cases were successfully discharged, and anti-inflammatory drugs were continued after the discharge. The postoperative follow-up period was 3–29 months. The re-examination of CTA suggested that the patency of *in situ* repair or reconstruction vessels was good, no rumen dilatation and severe stenosis were observed, and no abdominal hernia occurred.

In case 4, the patient had sudden abdominal pain 12 days after surgery and underwent emergency surgical treatment, during which bleeding from the abdominal aortic anastomosis was found, and emergency endovascular graft exclusion of the abdominal aorta was performed. Postoperatively, the patient developed chills and hyperpyrexia, and blood cultures showed gram-positive cocci and gram-negative bacilli. He developed massive hematemesis 16 days after the second operation and subsequently died of respiratory arrest, and the cause of death was considered to be the re-rupture of the aneurysm due to bacterial infection. In case 5, the duodenal fistula was damaged after the operation, a large amount of digestive juice was drained, and the patient died of abdominal aortic rupture and bleeding 1 week after the operation. The cause of death was considered to be that the reconstructed abdominal aorta was corroded by digestive juice resulting in a recurrence of rupture.

## Discussion and conclusions

Infected aneurysms are caused by bacteria or fungi entering vascular lesions or those growing on the intima of blood vessels through hematogenous spread, lymphatic transmission, infection of adjacent tissues around blood vessels, and so on **(author?)** (5). The chief predisposing factors are acquired or innate immunodeficiency, intravascular drug use, and pre-existing defects in the arterial wall. The most common pathogens causing infectious aneurysms are *Staphylococcus aureus, Salmonella, Streptococcus*, and *Escherichia coli*, and in some specific cases, *Staphylococcus epidermidis, Klebsiella, Haemophilus influenzae*. *Mycobacterium tuberculosis* can also cause infectious aneurysms (6). However, 14–40% of patients diagnosed with an infective aneurysm still do not have an identified causative organism (7). Contrast-enhanced CT is the most commonly used imaging method for early detection of infectious aneurysms, characterized by irregularly shaped aneurysms, which may be polycystic, with gas shadows or low-density shadows in the soft tissues around the aorta and irregular peripheral enhancement of the arterial wall.

Surgical treatment of infective aneurysms often involves open surgical repair of the infected segmental artery, extensive removal of the surrounding tissue, arterial revascularization by using an orthotopic graft replacement or extra-anatomic bypass (EAB), followed by an aggressive antibiotic therapy. Although surgical open surgery has long been considered an effective treatment for infected aneurysms, there is no consensus on *in situ* graft replacement or EAB for arterial revascularization. In addition, the use of foreign grafts in infected areas violates the basic principles of surgery and is a controversial strategy (8–10).

Commonly used grafts in surgery are Dacron or polytetrafluoroethylene vascular prostheses soaked in antibiotics, bovine pericardium, cryopreserved allografts, and autologous superficial femoral and jugular veins (11–14). Batt et al. (15) conducted a meta-analysis of patient age and reported the presence of prosthetic duodenal fistula (PDF). They also showed that the presence of virulent or non-virulent microbial factors indicated that combined *in situ* reconstruction of each factor was superior to extra-anatomic bypass reconstruction of the arterial blood supply. In a comparative analysis of cryopreserved allografts, autologous veins, and vascular grafts (silver polyester; rifampin) for infected aneurysms treated through *in situ* reconstruction surgery, the latter showed the most severe reinfection rate. Autologous veins had the lowest reinfection rate, while allografts had a higher postoperative anti-infective ability than that of the vascular grafts (16). It has been reported that the use of biological grafts is a reliable option for open repair of infected aneurysms because of its anti-infective ability and high survival rate (17). Heinola et al. (18) reported 1- and 5-year postoperative survival rates of 83 and 71%, respectively, for patients with infected aneurysms treated by using biological grafts. For larger vessels, the autologous vein does not have a sufficient caliber and resistance to perform revascularization. It is also difficult to operate when the autologous vein is sampled. Moreover, the operation time is too long for elderly patients to tolerate (19, 20). The use of allografts shortens the operation time and reduces surgical trauma. However, availability, cost, and graft-related complications (aneurysmal degeneration, dilatation, rupture, and reinfection) caused by graft degradation have limited the widespread use of allografts.

Sarac et al. (21) successfully repaired the aortic stump using autologous fascia-peritoneum tissue with abdominal aortic graft infection. They were also able to avoid postoperative rupture of the aortic stump. Embryologically, the peritoneum and fascia are derived from the cells similar to the endothelial and adventitial layers of blood vessels, respectively. The peritoneum develops from the mesoderm comprising mesothelial cells and connective tissue and contains blood vessels, lymphatic vessels, and nerves (22). Its adipose tissue contains a large number of mesenchymal stem cells and can differentiate into other cell types. Peritoneal mesothelial cells also have a certain degree of plasticity. These characteristics allow the use of the peritoneum for regenerative therapy (23, 24). The rectus fascial layer is composed of collagen. It is a tough connective tissue with good resistance to stress (21). The autologous fascia-peritoneum tissue is a highly viable and easily accessible tissue. Hence, it is an ideal vascular substitute. Pacholewicz et al. (25) successfully used the autologous fascia-peritoneum tissue as a patch on the pericardium of mongrel dogs and achieved satisfactory results as a pericardial substitute. They also demonstrated the antithrombotic properties of the peritoneum. Sarac et al. (26) demonstrated the ability of the fascia-peritoneum tissue to withstand continuous arterial pressure, good compliance, and resistance to intimal hyperplasia. García-Graz et al. (27) conducted animal experiments and showed that the fascia-peritoneum with posterior rectus sheath has good permeability and antithrombotic properties as a vascular graft.

The Chin et al. (28) study published the first autogenous peritoneo-fascial for repair after partial resection of the inferior vena cava in six patients; in 2013, the same team republished a study of 22 patients who underwent autogenous peritoneo-fascial transplantation to reconstruct the inferior vena cava (29). In the late follow-up of both studies, the conclusions were similar and the ideal surgical results were obtained. Dokmak et al. (30) showed that autogenous peritoneo-fascial tissue was also used to repair other abdominal veins, such as the hepatic and portal veins. Emmiler et al. (31) showed repair of vascular injuries following abdominal trauma with autogenous peritoneo-fascial tissue and since then provided a new treatment modality for vascular injury repair. In the study by Chin et al. (28) the authors highlight the advantages of the autogenous peritoneo-fascial patch in the repair of partial inferior vena cava resection, which has better flexibility, usability, reduces the risk of infection and thrombosis, is available in a short time and costs less, and can also be used immediately in emergencies compared to traditional repair techniques.

Antibiotics play a crucial role in the treatment of patients with infectious aneurysms, although the duration of antibiotic application is a matter of debate (32). An appropriate preoperative antibiotic therapy can not only control the inflammation, but can also effectively reduce periaortic adhesions, more easily debride perivascular infected tissue, and increase the success rate of orthotopic vascular graft replacement (1, 33). Kritpracha et al. (34) reported on 21 patients with infective aneurysms who were treated preoperatively with antibiotics for an average period of 10 days and reported a 30-day patient mortality rate of 19%. Sedivy et al. (35) preoperatively treated 32 patients with infective aneurysms using antibiotics for an average period of 16 days, and reported a 30-day patient mortality rate of 18%. Clough et al. (36) treated 11 patients with infective aneurysms using preoperative antibiotics for an average period of 42 days; the 30-day mortality rate was 11.1%. Di et al. (37) showed that the duration of preoperative antibiotic therapy was strongly associated with the 30-day mortality. For the duration of post-operative antibiotic therapy, most studies recommend at least 6 weeks of post-operative intravenous or oral antibiotic therapy (36, 38); for patients with abnormal inflammatory markers, a longer antibiotic therapy or even a lifelong oral antibiotic therapy is recommended (39, 40).

Among our 7 patients with infected aneurysms, 5 had infected abdominal aortic aneurysm, 1 had infected common iliac artery aneurysm, and the remaining 1 had infected internal iliac artery aneurysm. Because there was no suitable autologous vein for repair, we used autologous fascia-peritoneum with a posterior rectus sheath to repair or reconstruct the infected vessels *in situ*. The infected abdominal aortic aneurysms in 3 patients were repaired with an autologous fascia-peritoneum tissue patch, while the remaining 2 cases were treated with the autologous fascia-peritoneum tissue suture component forked *in situ* to reconstruct the abdominal aorta-bilateral iliac arteries. Two patients with infected iliac aneurysms were treated with autologous fascia-peritoneum tissue suture to reconstruct *in situ* tubular iliac arteries. All of them were covered with the greater omentum after reconstruction and were adequately irrigated and drained after surgery. Perioperatively, all patients underwent long-term antibiotic therapy based on the bacterial culture and susceptibility results of the infected tissue and blood. All 7 patients had a successful operation. Two patients died of anastomotic infection and massive hemorrhage after surgery; the remaining 5 patients survived. The patients were followed up for 2–19 months. The postoperative enhanced CT scan showed that the reconstructed artery was smooth, without any obvious stenosis or dilatation, and no abdominal hernia occurred.

Autologous fascia-peritoneum with a posterior rectus sheath can fight infections, in contrast to foreign grafts. In our study, 5 patients survived with a good survival rate. When autologous veins or foreign grafts cannot meet the surgical needs, they become a viable arterial substitute, which also provides a feasible treatment for refractory infectious aneurysms. However, long-term follow-up and large-scale clinical data are still needed to further confirm the feasibility of the treatment.

## Data availability statement

The original contributions presented in the study are included in the article/supplementary material, further inquiries can be directed to the corresponding authors.

## Ethics statement

The studies involving human participants were reviewed and approved by the Ethics committee of the Yantai Yuhuangding Hospital. The patients/participants provided their written informed consent to participate in this study. Written informed consent was obtained from the individual(s) for the publication of any potentially identifiable images or data included in this article.

## Author contributions

FS and MY: conception and design and revision of manuscript. WN, ZP, JZ, and HC: collection and assembly of data. LL, GL, and BY: data analysis and interpretation and manuscript writing. All authors contributed to the article and approved the submitted version.

## Conflict of interest

The authors declare that the research was conducted in the absence of any commercial or financial relationships that could be construed as a potential conflict of interest.

## Publisher's note

All claims expressed in this article are solely those of the authors and do not necessarily represent those of their affiliated organizations, or those of the publisher, the editors and the reviewers. Any product that may be evaluated in this article, or claim that may be made by its manufacturer, is not guaranteed or endorsed by the publisher.
